# Using Observational Data to Estimate the Effect of Hand Washing and Clean Delivery Kit Use by Birth Attendants on Maternal Deaths after Home Deliveries in Rural Bangladesh, India and Nepal

**DOI:** 10.1371/journal.pone.0136152

**Published:** 2015-08-21

**Authors:** Nadine Seward, Audrey Prost, Andrew Copas, Marine Corbin, Leah Li, Tim Colbourn, David Osrin, Melissa Neuman, Kishwar Azad, Abdul Kuddus, Nirmala Nair, Prasanta Tripathy, Dharma Manandhar, Anthony Costello, Mario Cortina-Borja

**Affiliations:** 1 UCL Institute for Global Health, London, United Kingdom; 2 Centre for Sexual Health and HIV Research, UCL Institute of Epidemiology and Health Care, London, United Kingdom; 3 Centre for Public Health Research, Massey University, Wellington, New Zealand; 4 Department of Medical Sciences, Cancer Epidemiology Unit, CeRMS and CPO-Piemonte, University of Turin, Turin, Italy; 5 Population, Policy and Practice Programme, UCL Institute of Child Health, London, United Kingdom; 6 Perinatal Care Project (PCP), Dhaka, Bangladesh; 7 Ekjut, Chakradharpur, Jharkhand, India; 8 Mother and Infant Research Activities (MIRA), Kathmandu, Nepal; Oslo University Hospital, Ullevål, NORWAY

## Abstract

**Background:**

Globally, puerperal sepsis accounts for an estimated 8–12% of maternal deaths, but evidence is lacking on the extent to which clean delivery practices could improve maternal survival. We used data from the control arms of four cluster-randomised controlled trials conducted in rural India, Bangladesh and Nepal, to examine associations between clean delivery kit use and hand washing by the birth attendant with maternal mortality among home deliveries.

**Methods:**

We tested associations between clean delivery practices and maternal deaths, using a pooled dataset for 40,602 home births across sites in the three countries. Cross-sectional data were analysed by fitting logistic regression models with and without multiple imputation, and confounders were selected a priori using causal directed acyclic graphs. The robustness of estimates was investigated through sensitivity analyses.

**Results:**

Hand washing was associated with a 49% reduction in the odds of maternal mortality after adjusting for confounding factors (adjusted odds ratio (AOR) 0.51, 95% CI 0.28–0.93). The sensitivity analysis testing the missing at random assumption for the multiple imputation, as well as the sensitivity analysis accounting for possible misclassification bias in the use of clean delivery practices, indicated that the association between hand washing and maternal death had been over estimated. Clean delivery kit use was not associated with a maternal death (AOR 1.26, 95% CI 0.62–2.56).

**Conclusions:**

Our evidence suggests that hand washing in delivery is critical for maternal survival among home deliveries in rural South Asia, although the exact magnitude of this effect is uncertain due to inherent biases associated with observational data from low resource settings. Our findings indicating kit use does not improve maternal survival, suggests that the soap is not being used in all instances that kit use is being reported.

## Background

Reducing maternal deaths during pregnancy, childbirth and the first 42 days after delivery is a major global health challenge addressed by the fifth Millennium Development Goal (MDG). The MDG target is to reduce the Maternal Mortality Ratio (MMR) by three-quarters between 1990 and 2015.[1] Ninety percent of such maternal deaths occur in Sub-Saharan Africa and South Asia. In South Asia, MMR declined 4% per year between 1990 and 2011.[2,3] In 2011, Bangladesh’s MMR was estimated at 247 per 100 000 live births (Uncertainty interval (UI) 197–309), India’s at 187 (UI 142–238), and Nepal’s at 316 (UI 241–407).[3]

Puerperal sepsis is an infection arising from the genital tract that can occur between rupture of membranes and 42 days after birth.[4] It is responsible for approximately 10% of maternal deaths in Africa and 12% in Asia.[5] Morbidity due to puerperal sepsis is estimated to affect between 5% and 10% of pregnant women.[6] However, obtaining cause-specific maternal morbidity and mortality estimates for low- and middle-income countries is notoriously difficult.[7,8] As an example most data comes from hospital-based studies that are not representative of the substantial proportion of deliveries that still occur in the home.[7] It is also difficult for mathematical and statistical models to derive accurate measurements for overall maternal mortality estimates when studies that report maternal deaths are uncommon.[8] Adding to this uncertainty, a hospital-based study in Mozambique showed sensitivities of less than 50% for a clinical diagnosis of infection-related maternal death when compared to the gold standard of diagnosis through autopsy.[9] Sepsis-related maternal deaths and morbidity are under-diagnosed and sepsis exacerbates risk from other causes of death such as haemorrhage and abortion.[10]

To prevent sepsis, the World Health Organization (WHO) promotes the observance of “six cleans” at the time of delivery: clean hands, clean perineum, clean delivery surface, clean cord and tying instruments, and clean cutting surfaces.[11] Clean delivery kits usually include soap for washing the birth attendant's hands and mother's perineum, a plastic sheet to provide a clean delivery surface, a clean thread for tying the umbilical cord, a new blade for cutting the cord, and pictorial instructions to illustrate the sequence of events during a delivery.[11]

A recent systematic review examined the effects of clean delivery kits on maternal and neonatal health with three studies testing the impact of complex intervention packages, including clean delivery kits, on maternal outcomes[12–15] Results from these studies indicate that clean delivery practices, especially the use of clean kits, improve the maternal outcome of puerperal sepsis.[13–15] However, the systematic review concluded that providing kits to facilitate clean delivery practices seemed commonsense, but none of the available studies provided evidence of independent effects of kits separable from those achieved by broader intervention packages.[12]

Given the known importance of clean delivery practices for maternal health, conducting cluster randomized control trials (cRCTs) testing their promotion either as a package (through clean delivery kits, for example) or individually would be unethical. However, examining the associations of clean delivery practices with maternal deaths using observational data allows estimating the potential impact that their successful promotion might have on maternal mortality at population level. To date, there has been a lack of high quality studies examining the effects of clean delivery practices on maternal mortality whilst accounting for biases using appropriate sensitivity analyses.

In this paper we use a large observational dataset from the control arms of four previously conducted cRCTs[16–19] to examine the associations between maternal mortality and the use of a clean delivery kit and hand washing with soap by the birth attendant in rural South Asian communities.

## Methods

### Study populations

We used data from 40,602 home deliveries in the control arms of four community-based cRCTs carried out between 2000 and 2011 in India, Bangladesh and Nepal.[16–20] In India, baseline data collected prior to the cRCT using the same data collection methods were also included. In Nepal, data collection continued after the completion of the cRCT and before the intervention was implemented in control clusters, allowing for the use of additional data from control clusters.

**Fig 1:**
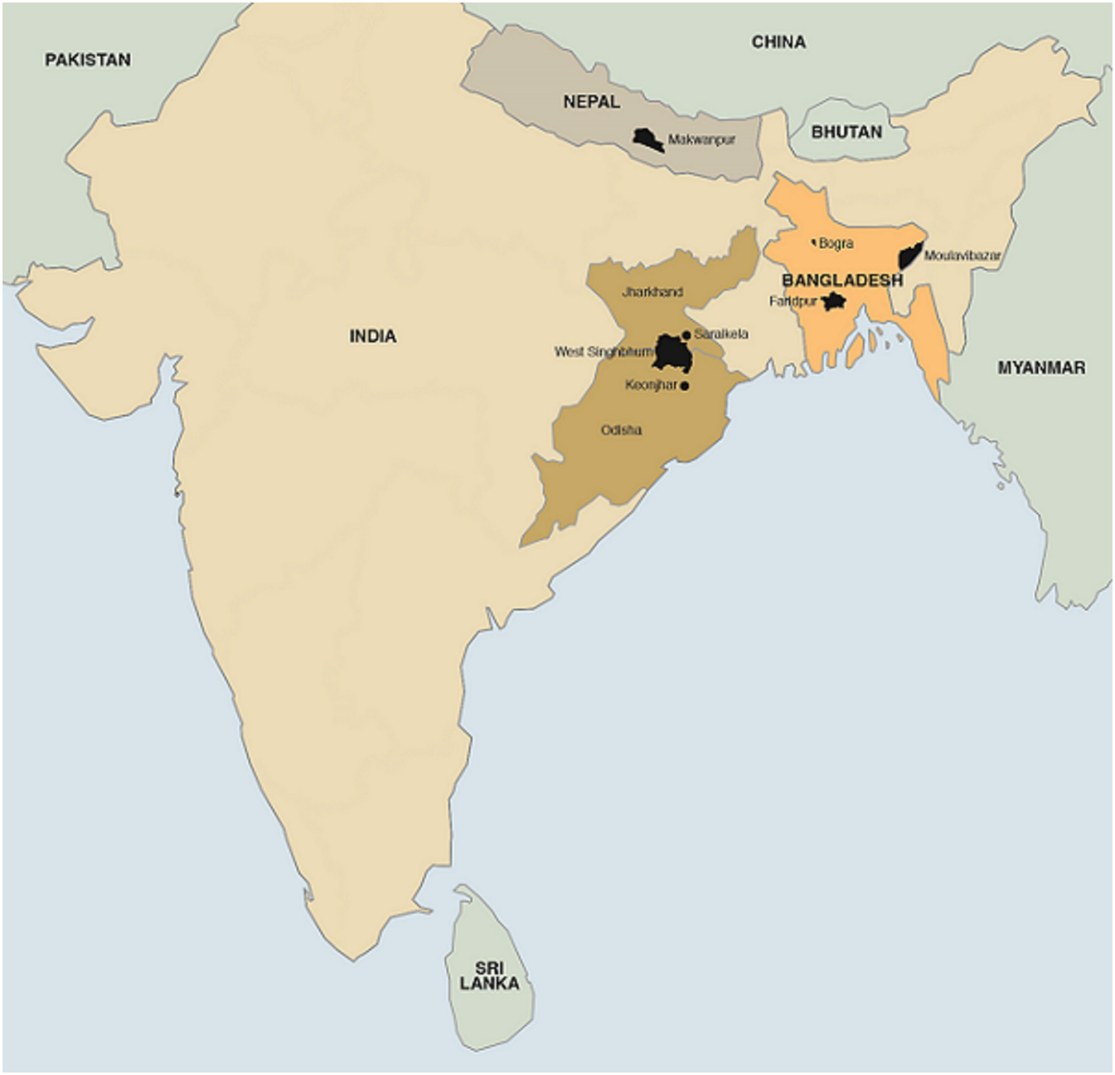
Location of sites included in the study, copyright UCL Medical Illustration Services.

The study areas included three rural districts in eastern India, three in Bangladesh and one in Nepal; Fig 1 shows their locations. In India and Nepal, clean delivery practices including kits were promoted and distributed through the health system as part of government initiatives to improve birth outcomes. In Bangladesh, BRAC, a developmental organisation, makes and distributes kits at a low cost. A previous publication reports details of kit manufacturing and distribution.[21] Data on kit use and hand washing were collected in each of the studies. Our analysis was limited to mothers of either live-born or stillborn infants delivered at home.

### Ethics statement

Research ethics approval for the trials during which data for the study came from, included the following in-country Ethical Review Committees (ERC): the ERC of the Diabetic Association of Bangladesh (BADAS); an independent ERC in Jamshedpur, India, steered by the Indian Council of Medical Research (ICMR) Guidelines of 2006 (Ekjut trial); and the Nepal Health Research Council. Approval was also obtained from the Institute of Child Health and Great Ormond Street Hospital for Children (UK) Research Ethics Committee.

Participants in the trials were all women of reproductive age (defined as aged 15–49) who had recently experienced a pregnancy and delivery. Although some of these participants would have been minors (defined as under 18 years of age), in South Asia residence tends to be patrilocal, so that a wife resides with her husband’s parents or at least in their locality. Obtaining parental consent for brides aged less than 18 years of age, was not possible in a context where they were judged old enough to live away from their parents, be married, and have children. Given younger women are known to be at increased risk of poorer health outcomes in delivery, it was decided to include participants who are less than 18 years of age in this secondary data analysis. Both the UCL and local ethics committees were aware of the age range and did not raise specific objection to this. Consent for minors was therefore the same as for older participants. All trials were conducted in disadvantaged areas with low levels of female literacy and all participants gave consent in writing or by thumbprint.

### Surveillance systems: data collection and management

Data were collected on paper, checked by auditors, entered by data entry operators and cross-checked by data managers. Databases were created and managed in Microsoft Access or SQL Server. Separate datasets for each study and a pooled dataset consisting of information common to the three sites were prepared for statistical analysis in Stata, release 12.0 (Stata Corp, College Station, Tx).[22] All sites used similar surveillance systems and gathered information about maternal socio-demographic characteristics and events during the antenatal, delivery and postnatal periods through a structured questionnaire administered to mothers around six weeks after delivery. Details of the individual surveillance systems can be found elsewhere.[16–20] All data included in this analysis can be found in S1 Data.

### Exposures and outcome

Table 1 describes the data collected by vital events surveillance systems that were similar in all three sites. Maternal death was defined by International Classification of Diseases, 10^th^ Revision (ICD-10) as death of a woman during pregnancy or up to 42 days after delivery or termination of pregnancy.[4] We were interested in the effect of hygiene during delivery, and therefore selected the main outcome as postpartum maternal death (after delivery and within 42 days). It was not possible to include a morbidity outcome such as puerperal sepsis as the main outcome, due to the unavailability of reliable markers. The exposures of interest were two intrapartum practices that could potentially reduce puerperal sepsis: use of clean delivery kit and hand washing with soap by birth attendant. As part of the survey questionnaire, women were specially questioned as to whether or not the birth attendant washed her hands with soap, prior to delivery.

**Table 1:** Characteristics of study populations included in the analysis.

Country	India	Bangladesh	Nepal
Location	Three districts of Jharkhand and Orissa (eastern India):	Three rural districts:	Makwanpur district
	Keonjhar, West Singhbhum and Saraikela	Bogra, Maulvibazaar and Faridpur	
Study period	1. Baseline surveillance: Nov 21, 2004—July 30, 2005	1. 1st cRCT: Feb 1, 2005 to Dec 31, 2007	1. cRCT: Nov 1, 2001 to Oct 31, 2003
	2. cRCT: July 31, 2005, to July 30, 2008	2. 2nd cRCT: Jan 1 2009 to June 20111	2. Surveillance data: Nov 1, 2003—March 2005
Study design	1. Baseline surveillance, not a cRCT	1. Factorial design, cluster randomised controlled trial, open cohort.	Cluster randomised controlled trial, matched design and closed cohort.
	2. Cluster randomised controlled trial, open cohort.	2. Cluster randomised controlled trial, open cohort	Post cRCT, roll-out of intervention into control clusters.
Cluster characteristics	8–10 villages with residents classified as tribal or OBC	Villages making up a union	Village Development Committees
Clusters analysed, n	18	9	12
Participants	Women aged between 15 and 49 who had given birth in study period, and their infants	Women aged between 15 and 49 who had given birth in study period, and their infants.	Women aged between 15 and 49, married and with potential to become pregnant in study period, and their infants
Deliveries analysed, n	11,063	25,591	3948
Maternal mortality rate prior to initial intervention (per 1000 00 live births)	510 [21]	380 [46]	539 [47]
Contents of clean delivery kits	Soap, razor, plastic sheet, string, gauze. Instructions available in government kits only.	Soap, razor, plastic sheet, string, gauze. Instructions available in government kits only.	Soap, razor, plastic sheet, string, gauze. Plastic coin to use as surface to cut the cord. Instructions available in government kits only.
Individual clean delivery practices recorded separately from kit use	Hand washing, use of boiled blade to cut cord, type of cord care (dry or other), use of boiled thread to tie the cord, use of plastic sheet and use of gloves.	Hand washing, use of boiled blade to cut cord, type of cord care (dry or other), use of boiled thread to tie the cord, use of plastic sheet and use of gloves.	Hand washing, use of boiled blade to cut cord, type of cord care (dry or other)
Concurrent activities to promote clean delivery practices and kit use	In both intervention and control areas, strengthening the activities of village health and sanitation committees.	Training was provided to nurses, doctors and paramedical staff in essential newborn care, including the six cleans.	Health service strengthening across intervention and control areas included training of all health workers on the six cleans.

### Confounders

Based on existing literature, the following potential confounders were considered: maternal age (15–49), maternal education (none, primary, and secondary and above), number of antenatal care visits (0–4+), delivery assisted by a skilled birth attendant (country-specific definitions defined by Demographic Health Survey data, most recent version for country in question: India and Nepal: doctor, nurse or trained midwife; Bangladesh: doctor, nurse, trained midwife, family welfare visitor, community skilled birth attendant),[23–25] household assets (all assets included households with the following items; television, fridge, electricity; some assets referred to households the following; a bicycle, radio, fan or phone, and no assets referred to a household not having any of the above mentioned assets), parity (0–4+), and study site.[7,26] The use of a clean delivery kit was considered a potential confounder in analyses exploring the effects of hand washing on maternal death. Initially, univariable analyses were performed to assess whether potential confounders, clean delivery practices and maternal mortality differed between deliveries with and without hand washing by birth attendant, separately for each study site (Table 2).

**Table 2:** Comparison of deliveries with and without hand washing.

Factors associated with hand washing	India				Bangladesh				Nepal			
	Overall	Hand washing	No hand washing	*p* ^*a*^	Overall	Hand washing	No hand washing	*p* ^*a*^	Overall	Hand washing	No hand washing	*p* ^*a*^
	*(n = 10*,*399)*	*(n* = 2677)	(*n* = 7722)		*(n = 21*,*952)*	(*n* = 17,639)	(*n* = 4313)		(*n* = 2309)	(*n* = 1258)	(*n* = 1051)	
**Postpartum maternal death, *n* (%)**												
No	10381 (99.83)	2676 (99.96)	7705 (99.78)	0.05	21919 (99.85)	17617 (99.88)	4302 (99.74)	0.048	2301 (99.65)	1254 (99.68)	1047 (99.62)	0.799
Yes	18 (0.17)	1 (0.04)	17 (0.22)		33 (0.15)	22 (0.12)	11 (0.26)		8 (0.35)	4 (0.32)	4 (0.38)	
**Use of clean delivery kit, *n* (%)**												
No	8750 (84.14)	1907 (71.24)	6843 (88.62)	<0.001	18283 (83.29)	14230 (80.67)	4053 (93.97)	<0.001	387 (16.76)	253 (20.11)	134 (12.75)	<0.001
Yes	1599 (15.45)	743 (25.75)	856 (11.09)		3472 (15.82)	3225 (18.28)	247 (5.73)		139 (6.02)	133 (10.57)	6 (0.57)	
Missing	50 (0.48)	27 (1.01)	23 (0.30)		197 (0.90)	184 (1.04)	13 (0.30)		1783 (77.22)	872 (69.32)	911 (86.68)	
**Maternal characteristics**												
Maternal education, *n* (%)												
No education	7797 (74.98)	1783 (66.60)	6014 (77.88)	<0.001	6013 (27.39)	4467 (25.32)	1546 (35.85)	<0.001	1967 (85.19)	1007 (80.05)	960 (91.34)	<0.001
Primary	525 (5.05)	101 (7.13)	334 (4.33)		7967 (36.29)	6302 (35.73)	1665 (38.60)		240 (10.39)	165 (12.12)	75 (7.14)	
Secondary	2077 (17.79)	703 (26.26)	1374 (17.79)		7968 (36.29)	6867 (38.93)	1101 (25.53)		102 (4.42)	86 (6.84)	16 (1.52)	
Missing	0 (0.00)	0 (0.00)	0 (0.00)		4 (0.02)	3 (0.02)	1 (0.02)		0 (0.00)	0 (0.00)	0 (0.00)	
Maternal age in years, *n* (%)												
<20	1021 (9.82)	307 (11.47)	714 (9.25)	<0.001	3156 (14.38)	2596 (14.72)	560 (12.98)	<0.001	172 (7.45)	102 (8.11)	70 (6.66)	<0.001
20–29	5488 (52.77)	1538 (57.48)	3950 (51.15)		14238 (64.86)	11518 (65.30)	2720 (63.07)		1384 (59.94)	803 (63.83)	581 (55.28)	
30–39	2155 (20.72)	414 (15.47)	1741 (22.55)		4287 (19.53)	3314 (18.79)	973 (22.56)		612 (26.50)	293 (23.29)	319 (30.35)	
40+	109 (1.07)	25 (0.93)	84 (1.09)		267 (1.22)	207 (1.17)	60 (1.39)		141 (6.11)	60 (4.77)	81 (7.71)	
Missing	1626 (15.54)	393 (14.68)	1233 (15.97)		4 (0.02)	4 (0.02)	0 (0.00)		0 (0.00)	0 (0.00)	0 (0.00)	
Household assets, *n* (%)^b^												
All	1630 (15.67)	506 (18.90)	1124 (14.56)	<0.001	8275 (37.70)	7038 (39.90)	1237 (28.68)	<0.001	56 (2.43)	48 (3.82)	8 (0.76)	<0.001
Some	6557 (63.05)	1634 (61.04)	4923 (63.75)		5417 (24.68)	4355 (24.69)	1062 (24.62)		1009 (43.70)	582 (46.26)	427 (40.63)	
None	2212 (21.27)	537 (20.06)	1675 (21.69)		8260 (37.63)	6246 (35.41)	2014 (46.70)		1243 (53.83)	627 (49.84)	616 (58.61)	
Missing	0	0	0		0 (0.00)	0 (0.00)	0 (0.00)		1 (0.04)	1 (0.08)	0 (0.00)	
Parity, *n* (%)												
1	2340 (22.50)	684 (25.55)	1656 (21.45)	<0.001	6507 (29.64)	5504 (31.20)	1003 (23.26)	<0.001	266 (11.52)	159 (12.64)	107 (10.18)	<0.001
2	2410 (23.18)	654 (24.43)	1756 (22.74)		6318 (28.68)	5171 (29.32)	1147 (26.59)		481 (20.83)	291 (22.13)	190 (18.08)	
3	1878 (18.06)	519 (19.39)	1359 (17.60)		4201 (19.14)	3278 (18.58)	923 (21.40)		446 (19.32)	263 (20.91)	183 (17.41)	
4	3757 (36.13)	816(30.48)	2941 (38.09)		4923 (22.43)	3683 (20.88)	1240 (28.75)		1163 (48.33)	545 (43.32)	571 (54.33)	
Missing	14 (0.13)	4 (0.15)	10 (0.13)		3 (0.01)	3 (0.02)	0 (0.00)		0 (0.00)	0 (0.00)	0 (0.00)	
**Antenatal period**												
Number of antenatal care visits, *n* (%)												
0	3413 (32.82)	755 (28.20)	2658 (34.42)	<0.001	7931 (36.13)	5973 (33.86)	1958 (45.40)	<0.001	1533 (66.39)	755 (60.02)	778 (74.02)	<0.001
1	1471 (14.15)	386 (14.42)	1085 (14.05)		4768 (21.72)	3805 (21.57)	963 (22.33)		257 (11.13)	138 (10.97)	119 (11.32)	
2	2375 (22.84)	560 (20.92)	1815 (23.50)		3423 (15.59)	2844 (16.12)	579 (13.42)		189 (8.19)	116 (9.22)	73 (6.95)	
3	1528 (14.69)	452 (16.88)	1076 (13.93)		2584 (11.77)	2157 (12..23)	427 (9.90)		162 (7.02)	111 (8.82)	51 (4.85)	
4	1606 (15.44)	522 (19.50)	1084 (14.04)		3232 (14.72)	2850 (16.16)	382 (8.82)		168 (7.28)	138 (10.97)	30 (2.85)	
Missing	6 (0.06)	2 (0.07)	4 (0.06)		14 (0.06)	10 (0.06)	4 (0.09)		0 (0.00)	0 (0.00)	0 (0.00)	
Skilled birth attendant^c^												
No	9816 (94.39)	2259 (84.39)	7557 (97.86)	<0.001	21276 (96.92)	16987 (96.30)	4289 (99.44)	<0.001	2302 (99.70)	1253 (99.60)	1049 (99.81)	0.466
Yes	523 (5.03)	410 (15.32)	113 (1.46)		666 (3.03)	642 (3.64)	24 (0.56)		7 (0.30)	5 (0.40)	2 (0.19)	
Missing	60 (0.58)	8 (0.30)	52 (0.67)		10 (0.05)	10 (0.06)	0 (0.00)		0 (0.00)	0 (0.00)	0 (0.00)	

^a^ *p*-value obtained through chi squared statistic or Fisher’s exact test where appropriate

^b^ Household assets include the following definition for the different categories: all assets include those households containing any one of the following items; television, fridge, electricity; some assets refer households having any one of the following; a bicycle, radio, fan or phone, and no assets refer to a household not having any of the above mentioned assets.

^c^ Country specific definitions defined by Demographic Health Survey data (most recent version for country in question).[23–25] India and Nepal: doctor, nurse or trained midwife; Bangladesh: doctor, nurse, trained midwife, family welfare visitor, community skilled birth attendant

After univariable analyses, directed acyclic graphs (DAGs) were used to inform the statistical modelling of the relationships between each of the separate clean delivery practices, maternal mortality and potential confounders to ensure that the confounders selected were appropriate.[27] The DAGs supported the appropriateness of all selected confounders for inclusion in the models. Details of confounder selection can be found in S1 Text.

### Statistical methods

Analyses were performed as follows: first, logistic regression models were fitted to the pooled data to examine the association of individual clean delivery practices with maternal death, controlling for confounders available at all sites to ensure comparability of results. To determine the appropriateness of using a pooled dataset, an interaction term was introduced between each individual clean delivery practice and study site. A likelihood ratio statistic comparing fully adjusted models with the interaction term to similar models without the interaction term, confirmed the appropriateness of pooling data from the three study sites for the exposure of hand washing (*p* = 0.771) as well as kit use (*p* = 0.121). Secondly, these analyses were repeated separately for the three study sites. Finally, for all models, possible modifying effects of the confounders on the association between clean delivery practices and maternal mortality were tested by including a two-way interaction term where it was decided *a priori* that there was a plausible explanation for this effect.

The adjusted models estimating the effect of hand washing on maternal mortality experienced convergence problems when using the data from the Nepal site as well as the pooled dataset. This was mainly due to the small number of mothers who died after delivery, low uptake of skilled birth attendants, and large numbers of missing data on kit use in Nepal. As a result, skilled birth attendant and clean delivery kit were not included in any of the adjusted analyses estimating the effect of hand washing on maternal mortality. To provide some information on how excluding these confounders could have affected our estimates, a sensitivity analysis was performed whereby results were compared both with and without skilled attendant and clean delivery kit, separately and simultaneously, using data from India and Bangladesh. Results indicated no differences, when comparing adjusted models with skilled attendant and kit use (AOR, 0.45, 95% CI: 0.24–0.87) to adjusted models without skilled attendant and kit use (0.43, 0.22–0.84). The models estimating the effect of kit use on postpartum maternal mortality also experienced convergence issues for the same aforementioned reasons, and hence it was not possible to include the Nepal data in this part of the analysis.

As data were collected from 18 geographic clusters in India, nine in Bangladesh, and 12 in Nepal, maternal mortality could be correlated within clusters. The estimated intra-cluster correlation coefficient (ICC) was <0.0001 using the pooled dataset, as well as for the individual study sites, indicating that such correlation was minimal. We therefore fitted logistic regression models with fixed effect terms only. Variance inflation factors (VIF) showed no evidence of multicollinearity in any model.

### Sensitivity analyses

#### Missing data

We compared demographic, antenatal, and delivery characteristics, including clean delivery practices, maternal and neonatal outcomes, between respondents with recorded data on hand washing and those with missing data, using chi-squared and Fisher’s exact tests where appropriate. In India, data on hand washing were missing in 6% (*n* = 664), in Bangladesh 14% (*n* = 3639) and in Nepal 42% (*n* = 1639) of all deliveries. To reduce bias and loss of information due to missing data, we used multiple imputation by chained equations (MICE) as implemented in the MI command in Stata, under the assumption that data were missing at random (MAR).[28] Variables used in the MICE models consisted of the key outcome maternal death, previously mentioned confounders, and covariates found to be predictors of missingness that were not already considered, including obstetric haemorrhage.[29,30] Although it was not possible to include skilled birth attendant and kit use as confounders in the adjusted model, it was possible to include them as predictors of missingness in the MICE models. To test modest departures from MAR, a weighted sensitivity analysis using the Selection Model Approach was applied to our findings after multiple imputation.[31–33] Details of this analysis can be found in S2 Text.

#### Exposure misclassification bias

The accuracy of recall of the main exposures of clean delivery practices may depend on factors such as neonatal or maternal survival, as well as on different morbidity patterns experienced by mother and infant. We followed the methods developed by Lyles and Lin, in which estimated odds ratios (OR) accounting for misclassification rates of the main exposure, hand washing, were obtained fitting adjusted logistic regression models with appropriate weights based on assumed sensitivities and specificities; standard errors for these estimates were calculated using a jackknife procedure.[34] Analysis for misclassification bias was carried out in SAS version 9.3.[35] Details are in S2 Text.

## Results

### Study population

We analysed data from 40,602 mothers who gave birth at home between 2005 and 2011 in India (*n* = 11,063), Bangladesh (*n* = 25,591) and Nepal *(n* = 3948). In total, there were 73 maternal deaths just after delivery and up to 42 days postpartum across all study sites; 18 deaths in India (0.16% of deliveries), 43 deaths in Bangladesh (0.17%), and 12 deaths in Nepal (0.30%). Median maternal age was 25 years in India, 24 in Bangladesh and 26 in Nepal. In India, 5% (590/11063) of mothers had a home delivery assisted by a skilled birth attendant, compared with 3% (900/25591) in Bangladesh, and 0.2% (7/3948) in Nepal. Clean delivery kits were used in 15% of deliveries in India (1684/11 063) and Bangladesh (3901/25 591), but in only 4% of deliveries in Nepal (157/3948). There was substantial variation in the proportion of birth attendants washing their hands before delivery: in India it was 24% (2677/11 063), compared with 69% (17639/25 591) in Bangladesh, and 32% (1258/3948) in Nepal.

Table 2 compares deliveries with and without hand washing by the birth attendant, and excludes cases with missing hand washing. There was evidence that hand washing improved maternal survival in India and Bangladesh (*p* = 0.050 and *p* = 0.048, respectively), but not in Nepal (*p* = 0.799); however, in Nepal only eight maternal deaths with data on hand washing were reported and four maternal deaths had no information on hand washing. Clean delivery kit use was associated with birth attendant hand washing in all three study sites (*p*<0.001).

### Clean delivery practices and maternal mortality

Table 3 shows estimates from the unadjusted analysis, and Table 4 results from adjusted analyses before and after multiple imputations, exploring the associations between clean delivery practices and maternal mortality. The unadjusted pooled analysis showed that hand washing was associated with a 54% reduction in the odds of a postpartum maternal death (OR 0.46, 95% CI: 0.26–0.36) and adjusted analysis a 49% reduction in maternal deaths (AOR 0.51, 0.28–0.93). Use of clean delivery kit was not associated with reductions in postpartum maternal mortality in the unadjusted analysis (1.19, 0.60–2.36) nor in the adjusted analysis (1.26, 0.62–2.56).

**Table 3:** Unadjusted odds ratios for association between clean delivery kit use and hand washing, with maternal mortality.

Clean delivery practices	Pooled data^a^		India		Bangladesh		Nepal	
	Unadjusted OR (95% CI)	*p* ^b^	Unadjusted OR (95% CI)	*p* ^b^	Unadjusted OR (95% CI)	*p* ^b^	Unadjusted OR (95% CI)	*p* ^b^
Use of clean delivery kit ^d^	1.19 (0.60–2.36)	0.616	0.69 (0.16–2.30)	0.619	1.46 (0.67–3.18)	0.344	^c^	
Washing hands prior to delivery	0.46 (0.26–0.36)	0.010	0.17 (0.02–1.27)	0.084	0.49 (0.24–1.01)	0.053	0.83 (0.21–3.35)	0.799

^a^ Pooled analysis adjusted for study site.

^b^ Wald test.

^c^ Unknown due to all mothers who died having missing data on clean delivery kit use

^d^ Excludes Nepal data due to convergence issues

**Table 4:** Adjusted odds ratios (95% CI) for the association between clean delivery kit use and hand washing, with maternal mortality obtained from logistic regression models with and without multiple imputation.

Clean delivery practices	Model type	Pooled data		India		Bangladesh		Nepal	
		AOR (95% CI)	*p* ^a^	AOR (95% CI)	*p* ^a^	AOR (95% CI)	*p* ^a^	AOR (95% CI)	*p* ^a^
Use of clean delivery kit	Logistic regression ^b^ ^,^ ^e^	1.26 (0.62–2.56)	0.519	0.66 (0.15–2.93)	0.587	1.61 (0.71–3.68)	0.256	^f^	
	Multiple imputation ^c^ ^,^ ^e^	1.18 (0.62–2.24)	0.612	0.68 (0.15–2.99)	0.605	1.45 (0.63–3.30)	0.381	^f^	
Washing hands prior to delivery	Logistic regression ^b^	0.51 (0.28–0.93)	0.028	0.15 (0.02–1.11)	0.063	0.57 (0.27–1.23)	0.154	0.83 (0.19–3.56)	0.800
	Multiple imputation ^c^ ^,^ ^d^	0.48 (0.26–0.90)	0.022	0.15 (0.02–1.13)	0.066	0.58 (0.27–1.25)	0.162	0.91 (0.23–3.65)	0.898

^a^ Wald test.

^b^ Adjusted for maternal age, maternal education, parity, number of antenatal care visits, household assets, and for the pooled analysis, study site.

^c^ Multiple imputation models taking into account variables describe in b, as well as predictors of missingness including obstetric haemorrhage, and skilled birth attendant

^d^ Multiple imputation models also included clean delivery kit use as predictor of missingness.

^e^ It was not possible to include Nepal in the pooled analysis of kit use due to convergence issues caused by large numbers of missing/unknown data.

^f^ Model would not converge due large number of deliveries with missing/unknown data on kit use

### Sensitivity analysis

A comparison of deliveries with complete and missing information on hand washing by the birth attendant can be found in S1 Table. Results from MICE models accounting for missing data under the MAR assumption can be found in Table 4, and show that imputed estimates and estimates from the observed data were similar. Results from the sensitivity analysis testing the MAR assumption revealed that estimates moved towards the null, but still remained highly significant (Table 5).

**Table 5:** Adjusted odds ratios (95% CI) for different departures from the missing at random assumption (δ*), for the exposure variable of hand washing assuming greater probability of hand washing data being missing when hand washing did not occur.

δ ^a^	AOR ^b^ (95% CI)
0.40	0.574 (0.338–0.975)
0.30	0.573 (0.337–0.975)
0.20	0.572 (0.336–0.974)
0.15	0.568 (0.332–0.970)
0.10	0.554 (0.321–0.958)

^a^ δ is the log odds ratio of the probability of hand washing data being observed when hand washing occurred compared to when hand washing did not occur

^b^ Models have been adjusted by similar confounders and predictors of missingness as multiple imputation models found in Table 4.

The sensitivity analysis to assess whether the estimates from the complete-case analysis were subject to differential misclassification bias revealed that the strength of the association between hand washing and postpartum maternal death weakened where the AOR ranged between 0.68 (95% CI: 0.21–2.25) and 0.54 (0.27–1.15) (Table 6).

**Table 6:** Adjusted odds ratios (95% CI) for different combinations sensitivity (SE) and specificity (SP) values, assuming differential misclassification in the instance of maternal death and maternal survival of the exposure variable of hand washing.

Assumed SE (maternal death, maternal survival)	Assumed SP (maternal death, maternal survival)		
	0.89, 0.85	0.93, 0.89	0.97, 0.93
0.73, 0.86	0.67 (0.18–2.51)	0.68 (0.21–2.25)	0.69 (0.23–2.06)
0.90, 0.94	0.53 (0.24–1.20)	0.54 (0.27–1.15)	0.55(0.27–1.11)

## Discussion

Our pooled, complete-case analysis for study sites in India, Bangladesh, and Nepal indicated that hand washing by the birth attendant was associated with a 49% reduction in the odds of postpartum maternal death after adjustment for potential confounders. Use of a clean delivery kit was not associated with a reduction in maternal mortality at individual sites or in the pooled analysis.

The difference in findings between the association of kit use and maternal mortality compared to hand washing and maternal mortality needs further elaboration. Given one of the key components of a kit, is soap, it is reasonable to assume that if used appropriately, a similar association would be occur, as was found with hand washing. The fact that there was no significant association between kit use and maternal mortality suggests that a possible explanation is that soap was not used in all instances that kit use was reported to have been used. This interpretation is in agreement with a previous analysis of the associations between clean delivery kit use and neonatal mortality that found not all components of the clean delivery kit were being used.[21] Another qualitative study from Nepal found that kit users didn’t always read instructions and when they did, they had difficulty understanding them.[36]

Our findings need to be interpreted with caution due to limitations imposed by the use of observational data.[37] The analysis testing the sensitivity to the MAR assumption indicated that the association between hand washing and maternal death was an over-estimation of the true effect; however results were still highly significant. The sensitivity analyses taking into account differential misclassification for reporting of hand washing by the birth attendant demonstrated that even modest reductions in sensitivity and specificity weakened the estimates obtained from the complete-case analysis.

Studies using maternal mortality as the main outcome measure are uncommon, given it is a relatively rare event, requiring a large sample size. Although we calculated there was 100% power to determine the observed effect of hand washing by the birth attendant on maternal mortality at the 95% significance level, there was only 30% power to determine the observed effect with kit use on maternal mortality. The dangers of using a post-hoc power calculation have been well documented, however this estimate demonstrates even a modest reduction for a rare event such as maternal mortality, requires a sample size much larger than was available for this analysis.[38–41] It is also important to bear in mind that given the non-significant finding from our analysis indicating an increase in mortality with kit use, it seems unlikely that even an adequately powered study would show any benefit.

A possible explanation for the large reduction in the odds of a maternal death with hand washing by the birth attendant, is this variable was serving as a proxy indicator for a type of healthy behaviour that improved overall maternal survival. It is possible that participants who reported hand washing exhibited a collective group of behaviours that was difficult to measure. For example, an article discussing possible explanations for conflicting results between observational studies showing a reduction of cardiovascular disease, cancer and all-cause mortality with antioxidant use, compared to non-significant findings from a randomised controlled trial, suggested these differences were due to residual confounding caused by inadequate adjustment for the complexity of social and environmental exposures acting across the life course.[42] In the present study, data were cross-sectional in nature and it was therefore not possible to capture confounding variables that occurred throughout the mother’s life that could potentially influence the use of different clean delivery practices as well as the mother’s and infant’s outcome in delivery. However, if the reductions in the odds of a maternal death were entirely due to hand washing serving as a proxy measure for unobserved confounders, then we would have expected a similar finding with kit use, which was not the case.

Other evidence suggests that improved maternal survival due to hand washing by the birth attendant is irrefutable. In the 1840s, the Hungarian clinician Ignaz Semelweiss was the first to promote hand washing with a chlorine solution, leading to a subsequent decline in puerperal sepsis mortality rates from more than 900 to 300 per 1000 births.[43] Hand washing campaigns have also been shown to improve child health overall.[44] A systematic review found that hand washing with soap has the potential to reduce diarrhoeal disease by 42–47%, with the possibility of saving millions of lives if implemented and scaled up appropriately.[44] Another recent systematic review found that water, sanitation and hygiene (WASH) interventions, including hand washing promotion, have benefits for the growth of children under five.[45] Hygiene campaigns aimed at improving clean delivery practices may have similar benefits.

Previously, we found that kit use was associated with a reduction in neonatal mortality and that a combination of clean delivery practices was essential to this improvement.[21] Given the potential for kits to not only improve neonatal survival but also reduce maternal mortality and morbidity, careful consideration needs to be given to their contents and appropriate clean delivery practices. Kits may also be used as a vehicle for components to reduce other causes of maternal mortality, such as misoprostol, a drug known to be effective in reducing the incidence of postpartum haemorrhage.[46] However, it is essential not to discourage women from delivering in institutions while promoting the use of clean delivery kits.

Given the evidence base for hygiene in improving maternal mortality and morbidity associated with puerperal sepsis, the question of how to promote beneficial practices in underserved rural populations in South Asia is an important one. A recent meta-analysis involving seven cRCTs suggested beneficial effects on neonatal and maternal survival of an intervention involving community mobilisation through participatory women’s groups.[47] In the three trials where the intervention was most successful and data were available, clean delivery practices, including clean delivery kit use and hand washing by the birth attendant were more common in intervention than control clusters.[16,18,19] Working with community-based women’s groups may therefore have substantial benefits for maternal survival, partly by improving clean delivery practices during home births in settings where they are common.

Our study has several strengths: it draws on a large, population-based dataset with a shorter recall period than Demographic Health Surveys (i.e. six weeks vs. up to five years), features an additional indicator unavailable elsewhere for home births (hand washing), and gives careful consideration to potential sources of bias. Our findings demonstrate that improving hygiene through hand washing is likely to improve maternal survival following home births in rural settings in South Asia where there is minimal access to skilled birth attendants. However, the true effect if all forms of bias were removed is difficult to gauge, and is most likely weaker than the estimate from the complete case analysis.

## Supporting Information

S1 Data Dataset for analysis of clean delivery practices and maternal mortality. (XLS)

S1 Text Use of directed acyclic graphs to select confounders. (DOCX)

S2 Text Sensitivity analysis for missing data bias and misclassification bias. (DOCX)

S1 Table Comparison of deliveries with missing and complete data on hand washing by the birth attendant. (DOCX)
