# Supplementary material for: Using Observational Data to Estimate the Effect of Hand Washing and Clean Delivery Kit Use by Birth Attendants on Maternal Deaths after Home Deliveries in Rural Bangladesh, India and Nepal
Source: PLoS One. 2015 Aug 21;10(8):e0136152. doi: 10.1371/journal.pone.0136152 (PMC4546655; doi:10.1371/journal.pone.0136152)
Supplement: S1 Text — (DOCX) [file pone.0136152.s002.docx]

**SI 1 Text: Confounder selection**

Directed acyclic graphs (DAGs) were used to model the associations between selected confounders with each other, with the individual clean delivery practices (exposures), and with the outcome of post-natal maternal death. These DAGs then informed the statistical modelling of the relationship between each of the separate clean delivery practices and maternal mortality, taking confounders into account.[^1^](#_ENREF_1) In order to better approximate the causal relationships, the DAGs were modelled in relation to the pregnancy timeline from the pre-conception period to the post-natal period. Figure 1 shows the relationship between handwashing and post-partum maternal death and shows the appropriateness of all confounders. Figure 2 shows the relationship between using a clean delivery kit and post-partum maternal death and, contrary to Figure 1 that illustrates the inappropriateness of including individual clean delivery practices as potential confounders.


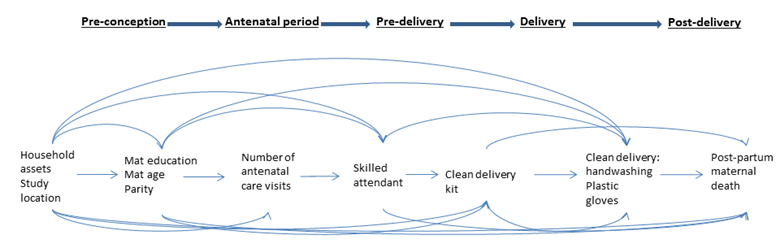


**Figure 1. DAG showing possible causal relationships between handwashing, maternal mortality, and potential confounders in relation to the pregnancy time-line**


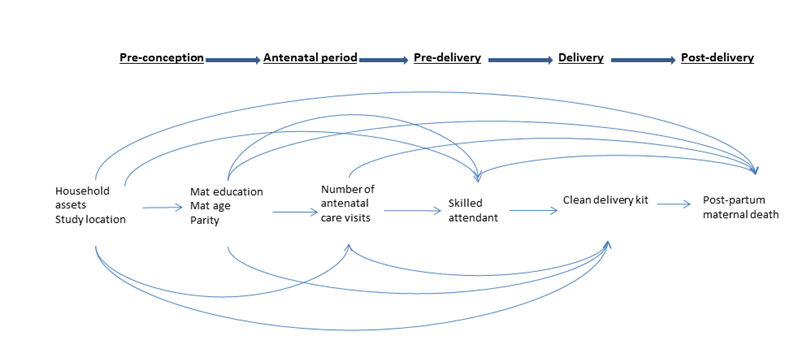


**Figure 2. DAG showing possible causal relationships between use of a clean delivery kit, maternal mortality, and potential confounders in relation to the pregnancy time-line**

**References:**

1. Textor J, Hardt J, Knuppel S. DAGitty: a graphical tool for analyzing causal diagrams. *Epidemiology (Cambridge, Mass)* 2011; **22**(5): 745.
