# Supplementary material for: Using Observational Data to Estimate the Effect of Hand Washing and Clean Delivery Kit Use by Birth Attendants on Maternal Deaths after Home Deliveries in Rural Bangladesh, India and Nepal
Source: PLoS One. 2015 Aug 21;10(8):e0136152. doi: 10.1371/journal.pone.0136152 (PMC4546655; doi:10.1371/journal.pone.0136152)
Supplement: S2 Text — (DOCX) [file pone.0136152.s003.docx]

**SI 2 Text: Sensitivity analysis**

*Missing data*

As previously described, multiple imputation by chained equations (MICE) models were used to impute the data, assuming data were missing at random (MAR). Due to differences between sites in the way data were collected, predictors of missing data, and the amount of missing data, the missing data mechanism might have differed between study sites; data were therefore imputed separately. Rubin’s rules were used to summarize estimates and their standard errors from analyses of 15 separate imputed datasets.[^1^](#_ENREF_1)

It is difficult to ascertain the missingness mechanism for the handwashing variable, especially given that our data comes from three separate study sites. Indeed, even in circumstances where the mechanism is not as ambiguous as is the case with this dataset, it is impossible to determine whether data is MAR or missing not at random (MNAR) from the data alone.[^2^](#_ENREF_2) When performing multiple imputation assuming the data is MAR estimates for handwashing are subject to bias if data is MNAR whether or not the missingness mechanism is dependent on the maternal death outcome.[^3^](#_ENREF_3)

To assess the sensitivity of our findings against modest departures from the MAR assumption, a weighted sensitivity analysis using the Selection Model Approach was applied.[^4-6^](#_ENREF_4) Briefly, once data had been imputed under MAR, parameter estimates from each imputed dataset were reweighted to allow for the data to be missing not at random (MNAR). The chosen weights, used to reweight the data to account for MNAR, are dependent on the assumed degree of departure from MAR. The parameter used to re-weight the data, denoted by δ, is the log odds ratio of the probability of handwashing data being observed when handwashing occurred compared to when handwashing did not occur.[^4-6^](#_ENREF_4) If δ=0, handwashing could be considered to be MAR, δ>0 indicates that the probability of observing handwashing when handwashing occurred was greater than when it did not, and δ<0 indicates that the probability of observing handwashing when handwashing occurred was less. As δ decreases from zero, the probability of handwashing data being observed when handwashing occurred is less than the probability of handwashing data being observed when handwashing did not occur (i.e. greater probability of missing handwashing variable when handwashing occurred). We hypothesize that due to the social desirability bias in reporting clean delivery practices, it is more likely that handwashing was missing in instances where handwashing was not used, compared to when handwashing was used (i.e. δ>0).

To gain insight into the missingness mechanism, logistic regression models were fitted to explore the relationship with missing handwashing, and potential predictors of missingness including maternal death. A multivariate model was fitted with the outcome of missing handwashing, and imputed values of potential predictors of missingness including the study outcome.[^6^](#_ENREF_6) Results indicated that the missingness mechanism depends on a neonatal death, clean delivery kit use, maternal age, and skilled delivery attendant. There was some evidence that the outcome of a maternal death was associated with missing handwashing data.

To test the stability of our model, we considered different degrees of departure from the MAR assumption by considering plausible values of δ ranging from 0.10 to 0.40. This range corresponds to odds ratios for the data being observed when handwashing occurred compared to when it did not, ranging from 1.11 to 1.50 (i.e. exponential of 0.10 and 0.40).

*Exposure misclassification bias:*

Maternal death was used as a proxy for which we determined how accurately handwashing by the delivery attendant was reported. In the event of a maternal death, there is likely to be reduced sensitivity and increased specificity in the ability to accurately report handwashing. As an example, in the event of a maternal death it is expected that a close relative will be searching for explanations as to why the death occurred, and that by under-reporting behaviours that improve survival they may partially explain why the death occurred, which will in turn decrease the sensitivity. Using the same reasoning, it is likely that specificity will be higher than when a woman survived, as most relatives are unlikely to classify handwashing as occurring, when in fact it did not occur, as they are searching for an explanation of why the woman did not survive.

In most cases, the mother will survive childbirth. The sensitivity of reporting handwashing in these cases is likely to be higher than in the event of a maternal death as mothers are going to be more likely to report desirable behaviours. Using the same reasoning, it is likely that the specificity will be lower than in the instance of a maternal death, as women are most likely to misclassify not washing their hands as washing their hands in order to report socially desirable behaviours.

Methods based on a weighted logistic regression model recently developed by Lyles and Lin allow estimating odds ratios accounting for misclassification rates of the main exposure.[^7^](#_ENREF_7) The required weights are obtained from the positive and negative predictive values, which are computed using pre-specified sensitivities and specificities, the outcome of interest, the observed exposure of interest and other important covariates. The weights are then used to fit the model of interest to an expanded dataset[^7^](#_ENREF_7) and a jackknife approach is used to compute standard errors for the estimated odds ratios.

For our analyses, we used a similar approach, assuming differential misclassification using complete-case analysis only. Our model included: the main exposure of handwashing, the outcome of maternal death, the confounders of study site and maternal age and the normalized weights. Due to complexities in assigning different weights to each level of the models’ parameters, only those confounders with the greatest effect on estimates evaluating for effects of handwashing on maternal mortality were included.

Differential misclassification assumes that sensitivities and specificities would differ depending on whether the mother lived or died. Based on this assumption, we tried several combinations of sensitivities and specificities to test the robustness of our findings, as shown in the Table 2. The restrictions imposed on the choice of different sensitivities and specificities were as follows:

*Probability of handwashing < sensitivity of handwashing*

*Probability of handwashing > 1- specificity of handwashing*

It was observed that 62% of delivery attendants were reported to have washed their hands, and this limited the extent to which we could evaluate different sensitivities and specificities.

**Table 2: Combinations of sensitivities and specificities used to evaluate misclassification bias**

|  | **Sensitivity** | **Specificity** | **Sensitivity** | **Specificity** |
| --- | --- | --- | --- | --- |
| **Maternal outcome** | **Maternal survival** | | **Post-partum maternal death** | |
| Combination 1 | 0.73 | 0.89 | 0.86 | 0.85 |
| Combination 2 | 0.73 | 0.93 | 0.86 | 0.89 |
| Combination 3 | 0.73 | 0.97 | 0.96 | 0.93 |
| Combination 4 | 0.90 | 0.89 | 0.84 | 0.85 |
| Combination 5 | 0.90 | 0.93 | 0.94 | 0.89 |
| Combination 6 | 0.90 | 0.97 | 0.94 | 0.93 |

**References:**

1. Rubin D. Multiple imputation for nonresponse in surveys. New York: John Wiley & Sons; 1987.

2. Potthoff, R, Tudor, G, Pieper, K, Hasselblad, V. Can one assess whether missing data are missing at random in medical studies? *Stat Methods Med Res* 2006; **15**(3): 213-34.

3. Spratt, M, Carpenter, J, Sterne, J, et al. Strategies for Multiple Imputation in Longitudinal Studies. *American Journal of Epidemiology* 2010; **172**(4): 478-87.

4. Carpenter J, Kenward M, White I. Sensitivity analysis after multiple imputation under missing at random: a weighting approach. *Stat Methods Med Res* 2007; **16**(3): 259-75.

5. Carpenter J, Pocock S, Lamm C. Coping with missing data in clinical trials: a model-based approach applied to asthma trials. *Stat Med* 2002; **21**(8): 1043-66.

6. Heraud-Bousquet, V, Larsen, C, Carpenter, J, Desenclos, JC, Le Strat, Y. Practical considerations for sensitivity analysis after multiple imputation applied to epidemiological studies with incomplete data. *BMC medical research methodology* 2012; **12**: 73.

7. Lyles R, Lin J. Sensitivity analysis for misclassification in logistic regression via likelihood methods and predictive value weighting. *Stat Med* 2010; **29**(22): 2297-309.
