# Supplementary material for: Using Observational Data to Estimate the Effect of Hand Washing and Clean Delivery Kit Use by Birth Attendants on Maternal Deaths after Home Deliveries in Rural Bangladesh, India and Nepal
Source: PLoS One. 2015 Aug 21;10(8):e0136152. doi: 10.1371/journal.pone.0136152 (PMC4546655; doi:10.1371/journal.pone.0136152)
Supplement: S1 Table — (DOCX) [file pone.0136152.s004.docx]

**SI 1 Table: Comparison between deliveries with complete information on handwashing and deliveries with missing information on handwashing**

| **Factors Associated with Handwashing** | **India** |  |  | **Bangladesh** |  |  | **Nepal** |  |  |  |
| --- | --- | --- | --- | --- | --- | --- | --- | --- | --- | --- |
|  | **Handwashing  present  (*n*=10 399)** | **Handwashing  missing  (*n*=664)** | ***p-*value ^a^** | **Handwashing  present  (*n*=21 952)** | **Handwashing  missing  (*n*=3639)** | ***p-*value ^a^** | **Handwashing  present  (*n*=2309)** | **Handwashing  missing  (*n*=1639)** | ***p-***  **value ^a^** | |
| **Maternal death *n* (%)** |  |  |  |  |  |  |  |  |  |  |
| No | 10381 (99.83) | 664 (100.00) | 0.623 | 21919 (99.85) | 3629 (99.73) | 0.090 | 2301 (99.65) | 1635 (99.76) | 0.771 |  |
| Yes | 18 (0.17) | 0 (0.00) |  | 33 (0.15) | 10 (0.27) |  | 8 (0.35) | 4 (0.24) |  |  |
| Missing | 0 (0.00) | 0 (0.00) |  | 0 (0.00) | 0 (0.00) |  | 0 (0.00) | 0 (0.00) |  |  |
| **Neonatal health** |  |  |  |  |  |  |  |  |  |  |
| Neonatal survival |  |  |  |  |  |  |  |  |  |  |
| Alive at 28 days | 9540 (94.38) | 611 (95.77) | 0.137 | 20796 (97.19) | 3420 (96.39) | 0.009 | 2157 (95.57) | 1591 (98.45) | <0.001 |  |
| Neonatal death | 568 (5.62) | 27 (4.23) |  | 602 (2.81) | 128 (3.61) |  | 100 (4.43) | 25 (1.55) |  |  |
| Stillbirth |  |  |  |  |  |  |  |  |  |  |
| No | 10108 (97.20) | 638 (96.08) | 0.094 | 21398 (97.48) | 3548 (97.50) | 0.935 | 2257 (97.75) | 1616 (98.60) | 0.054 |  |
| Yes | 291 (2.80) | 26 (3.92) |  | 554 (2.52) | 91 (2.50) |  | 52 (2.25) | 23 (1.40) |  |  |
| **Clean delivery practices** | 0 (0.00) | 0 (0.00) |  | 0 (0.00) | 0 (0.00) |  | 0 (0.00) | 0 (0.00) |  |  |
| Use of clean delivery kit, *n* (%) |  |  |  |  |  |  |  |  |  |  |
| No | 8750 (84.14) | 528 (79.52) | <0.001 | 18283 (83.29) | 3033 (83.35) | <0.001 | 387 (16.76) | 146 (8.91) | <0.001 |  |
| Yes | 1599 (15.38) | 85 (12.80) |  | 3472 (15.82) | 429 (11.78) |  | 139 (6.02) | 18 (1.10) |  |  |
| Missing | 50 (0.48) | 51 (7.68) |  | 197 (0.90) | 177 (4.86) |  | 1783 (77.22) | 1475 (89.99) |  |  |
| Use of plastic sheet, *n* (%) |  |  |  |  |  |  |  |  |  |  |
| No | 9580 (92.12) | 611 (92.02) | 0.005 | 10888 (49.60) | 1821 (50.04) | 0.011 | ^b^ | ^b^ | ^b^ |  |
| Yes | 819 (7.88) | 51 (7.68) |  | 11058 (50.38) | 1813 (49.82) |  | ^b^ | ^b^ |  |  |
| Missing | 0 (0.00) | 2 (0.30) |  | 6 (0.03) | 5 (0.16) |  | ^b^ | ^b^ |  |  |
| Use of gloves to assist delivery, *n* (%) |  |  |  |  |  |  |  |  |  |  |
| No | 10036 (96.51) | 610 (91.87) | <0.001 | 19679 (89.65) | 3234 (88.87) | <0.001 | ^b^ | ^b^ | ^b^ |  |
| Yes | 363 (3.49) | 54 (8.13) |  | 2198 (10.01) | 375 (10.31) |  | ^b^ | ^b^ |  |  |
| Missing | 0 (0.00) | 0 (0.00) |  | 75 (0.34) | 31 (0.82) |  | ^b^ | ^b^ |  |  |
| **Maternal characteristics** |  |  |  |  |  |  |  |  |  |  |
| Maternal education, *n* (%) |  |  |  |  |  |  |  |  |  |  |
| No education | 7797 (74.98) | 463 (69.73) | 0.009 | 6013 (27.39) | 863 (23.72) | <0.001 | 1967 (85.19) | 1461 (89.14) | 0.001 |  |
| Primary | 525 (5.05) | 44 (6.63) |  | 7967 (36.29) | 1339 (36.80) |  | 240 (10.39) | 124 (7.57) |  |  |
| Secondary | 2077 (19.77) | 157 (23.64) |  | 7968 (36.30) | 1436 (39.46) |  | 102 (4.42) | 54 (3.29) |  |  |
| Missing | 0 (0.00) | 0 (0.00) |  | 4 (0.02) | 1 (0.03) |  | 0 (0.00) | 0 (0.00) |  |  |
| Maternal age in years, *n* (%) |  |  |  |  |  |  |  |  |  |  |
| <20 | 1021 (9.82) | 92 (13.86) | <0.001 | 3156 (14.38) | 714 (19.62) | <0.001 | 172 (7.75) | 107 (6.53) | 0.008 |  |
| 20–29 | 5488 (52.77) | 317 (47.74) |  | 14238 (64.86) | 2315 (63.62) |  | 1384 (59.94) | 912 (55.64) |  |  |
| 30–39 | 2155 )20.72) | 100 (15.06) |  | 4287 (19.53) | 582 (15.99) |  | 612 (26.50) | 503 (30.69) |  |  |
| 40+ | 109 (1.05) | 5 (0.75) |  | 267 (1.22) | 27 (0.74) |  | 141 (6.11) | 117 (7.14) |  |  |
| Missing | 1626 (15.64) | 150 (22.59) |  | 4 (0.02) | 1 (0.03) |  | 0 (0.00) | 0 (0.00) |  |  |
| Household assets, *n* (%) |  |  |  |  |  |  |  |  |  |  |
| All | 1630 (15.67) | 117 (17.62) | 0.193 | 8275 (37.70) | 1406 (38.64) | 0.001 | 56 (2.43) | 31 (1.89) | <0.001 |  |
| Some | 6557 (63.05) | 422 (63.55) |  | 5417 (24.68) | 974 (26.77) |  | 1009 (43.70) | 600 (36.61) |  |  |
| None | 2212 (21.27) | 125 (18.83) |  | 8260 (37.63) | 1259 (34.63) |  | 1243 (53.83) | 1008 (61.50) |  |  |
| Missing | 0 (0.00) | 0 (0.00) |  | 0 (0.00) | 0 (0.00) |  | 1 (0.04) | 0 (0.00) |  |  |
| Parity, n (%) |  |  |  |  |  |  |  |  |  |  |
| 1 | 2340 (22.50) | 215 (32.38) | <0.001 | 6507 (29.64) | 1280 (35.17) | <0.001 | 266 (11.52 | 163 (9.95) | <0.001 |  |
| 2 | 2410 (23.18) | 139 (20.93) |  | 6318 (28.78) | 1065 (29.27) |  | 481 (20.83) | 290 (17.69) |  |  |
| 3 | 1878 (18.06) | 128 (19.28) |  | 4201 (19.14) | 620 (17.04) |  | 446 (19.32) | 268 (16.35) |  |  |
| 4 | 3757 (36.13) | 181 (27.26) |  | 4823 (22.43) | 674 (18.52) |  | 1116 (48.33) | 918 (56.1) |  |  |
| missing | 14 (0.13) | 1 (0.15) |  | 3 (0.01) | 0 (0.00) |  | 0 (0.00) | 0 (0.00) |  |  |
| **Antenatal period** |  |  |  |  |  |  |  |  |  |  |
| Number of antenatal care visits, *n* (%) |  |  |  |  |  |  |  |  |  |  |
| 0 | 3413 (32.82) | 198 (29.82) | 0.005 | 7931 (36.13) | 1274 (35.01) | 0.089 | 1533 (66.39) | 1228 (74.92) | <0.001 |  |
| 1 | 1471 (14.15) | 94 (14.16) |  | 4768 (21.72) | 813 (22.34) |  | 257 (11.13) | 163 (9.95) |  |  |
| 2 | 2375 (22.84) | 137 (20.63) |  | 3423 (15.59) | 626 (17.20) |  | 189 (8.19) | 104 (6.35) |  |  |
| 3 | 1582 (14.69) | 94 (14.16) |  | 2584 (11.77) | 401 (11.02) |  | 162 (7.02) | 92 (5.61) |  |  |
| 4 | 1606 (15.44) | 140 (21.08) |  | 3232 (14.72) | 521 (14.32) |  | 168 (7.28) | 52 (3.17) |  |  |
| Missing | 6 (0.06) | 1 (0.15) |  | 14 (0.06) | 4 (0.11) |  | 0 (0.00) | 0 (0.00) |  |  |
| **Delivery period** |  |  |  |  |  |  |  |  |  |  |
| Delivery by a skilled birth attendant |  |  |  |  |  |  |  |  |  |  |
| No | 9816 (94.39) | 595 (89.61) | <0.001 | 21276 (96.92) | 3397 (93.35) | <0.001 | 2302 (99.70) | 1639 (100.00) | 0.046 |  |
| Yes | 523 (5.03) | 67 (10.09) |  | 466 (2.12) | 234 (6.43) |  | 7 (0.30) | 0 (0.00) |  |  |
| Missing | 60 (0.58) | 2 (0.30) |  | 10 (0.05) | 8 (0.22) |  | 0 (0.00) | 0 (0.00) |  |  |
| Obstetric haemorrhage |  |  |  |  |  |  |  |  |  |  |
| No | 6392 (61.47) | 357 (53.77) |  | 14500 (66.05) | 2166 (59.52) | <0.001 | 2105 (91.17) | 1517 (92.56) | 0.118 |  |
| Yes | 352 (3.38) | 17 (2.56) |  | 7450 (33.94) | 1471 (40.42) |  | 204 (8.83) | 122 (7.44) |  |  |
| Missing | 3655 (35.15) | 290 (43.46) |  | 2 (0.01) | 3 (0.05) |  | 0 (0.00) | 0 (0.00) |  |  |

1. *p-*value obtain with a Wald test
2. b Data were not collected in the study
3. Country specific definitions defined by District Health Survey data (most recent version in question). India and Nepal: Doctor, Nurse or trained midwife; Bangladesh: doctor, nurse, midwife, paramedic, family welfare visitor, community skilled birth attendant
